# Phenotypic and Molecular Characterization of MCF10DCIS and SUM Breast Cancer Cell Lines

**DOI:** 10.1155/2013/872743

**Published:** 2013-01-16

**Authors:** Nandita Barnabas, Dalia Cohen

**Affiliations:** ^1^Asterand US, 440 Burroughs, Tech One Building, Suite 501, Detroit, MI 48202-3420, USA; ^2^ALN Associates, 170 Mount Vernon Street, Winchester, MA 01890, USA

## Abstract

We reviewed the phenotypic and molecular characteristics of MCF10DCIS.com and the SUM cell lines based on numerous studies performed over the years. The major signaling pathways that give rise to the phenotype of these cells may serve as a good resource of information when researchers in drug discovery and development use these cells to identify novel targets and biomarkers. Major signaling pathways and mutations affecting the coding sequence are also described providing important information when using these cells as a model in a variety of studies.

## 1. Introduction

Human tumor-derived cell lines grown *in vitro* and *in vivo* are important models to study cancer development, progression, and therapeutic response and resistance to anticancer drugs. However, for the last several years the relevance of cell lines in clinical cancer research has been criticized due to the properties of cell lines that differ when compared with primary tumor cells. New technologies such as next generation sequencing have enabled molecular characterization and identification of signaling pathways in specific cell lines. This characterization has facilitated the identification of cancer cell lines that are more clinically relevant for biological experiments as well as drug research and development. In addition, well characterized cells enable the identification of potential biomarkers for the development of companion diagnostics. Although there are still limitations with the relevance to the clinic, well characterized cancer cell lines will continue to be an important source for drug R&D and studying cancer biology. 

In this paper, we review relevant information available in numerous studies that encompass the characterization of the different breast cancer cell lines available at Asterand US. This review will be a valuable resource for researchers in academia and industry for the use of the relevant cell type in their research. The MCF10DCIS cell line was licensed to Asterand US by Wayne State University and the SUM cell lines were licensed to Asterand US by the University of Michigan. 

## 2. MCF10DCIS.com

**Figure 1:**
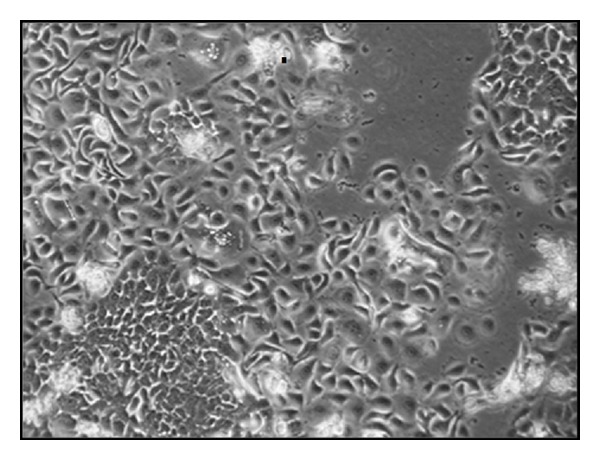
Morphology of MCF10DCIS and human breast cancer cell line in culture.

 MCF10DCIS.com is a clonal breast cancer cell line derived from a xenograft originating from premalignant MCF10AT cells that were injected into severe combined immune-deficient mice. The morphology of the MCF10DCIS cell line is shown in Figure 1. Injection of the MCF10DCIS cells into SCID mice resulted in rapidly growing lesions that are predominantly comedo ductal carcinoma *in situ*. Solid or comedo growth patterns are high grade ductal carcinoma* in situ* [1]. MCF10DCIS cells were shown to be reproducible from DCIS-like comedo lesions that spontaneously progress to IDC as xenografts in immunodefficent mice. Polizzotti and coworkers [2] used six (MCF10 series) *in vitro* cultures including MCF10DCIS to mimic the three grades of breast cancer along the metastatic cascade namely, nonmalignant, noninvasive carcinoma, and invasive carcinoma *in vivo*.

### 2.1. Key Signaling Proteins

CD44 is a cell-surface glycoprotein involved in cell-cell and cell extracellular matrix interactions including the migration and invasion of cancer cells and has been used as a key cancer stem cell-surface marker in various malignancies including breast cancer [3]. So et al. [4] showed growing malignant potential of MCF10 cell lines including MCF10DCIS with the decrease of expression of the standard 85-KDa isoform of CD44 and an increased expression of its 10–250 KDa variants, namely, CD44v, CD44v3, and CD44v6, and categorized them as markers of breast cancer progression. They have described a cascade of signaling proteins namely, Pak4, Stat3, pAkt, and pErk, in the MCF10 breast cancer model including two tumorigenic cell lines, MCF10DCIS and MCF10Ca1. High expression of CD44v can trigger the activities of several transmembrane receptor kinases [5]. Membrane receptors known to interact with CD44 and their downstream signals activate Stat3 leading to tumor progression and invasion [6]. Stat3 (signal transducer and activator of transcription 3) is part of a family known as the STAT genes. Normally, the Stat3 protein is switched on and off in response to signals that control cell growth and development. Abnormal Stat3 protein activation has been identified in many cancers that include breast, prostate, and pancreas, as well as cancers of blood-forming cells (leukemia and lymphoma). Excess Stat3 protein potentially contributes to the growth of cancers. Pak4 is a serine/threonine kinase and found to be highly expressed in breast cancer and associated with a signaling pathway leading to malignancy and formation of mammary tumors in nude mice [7]. It is highly expressed in MCF10DCIS compared to its precursor nontumorigenic cells lines [4]. Similarly they found high levels of phosphorylated forms of Erk and Akt namely pErk and pAkt were found only in the tumorigenic MCF10DCIS and MCF10CA1a cell lines indicating that overactivation of Erk and Akt is critical for developing malignant breast cancer. Erk and Akt are central protein kinases that mediate cellular responses to a diverse range of extracellular stimuli, including growth factors and cytokines, to regulate cell cycle progression and cell motility [8]. PAK (p21 activated kinase) belongs to a family of serine/threonine kinases that play a pivotal role in physiologic processes including motility, survival, invasion, and mitosis [9]. PAK's are widely expressed in a variety of tissues and are often upregulated or hyperactivated in a variety of human cancers including breast cancer [9, 10]. Upregulation of PAK1 and downregulation of HoxD10 were observed in MCF10DCIS cells [11]. HoxD10 is a homeobox transcription factor mediating gene responsible for the inhibition of invasiveness of cancer cells [12]. In luminal breast cancer, the expression and localization of PAK1 protein were assessed in primary tumors from 403 premenopausal patients that were randomized for two years of adjuvant tamoxifen or no treatment. Elevated expression and/or nuclear localization of PAK1 were associated with resistance to tamoxifen therapy [13].

### 2.2. **PI3K  Mutation**

**Figure 2:**
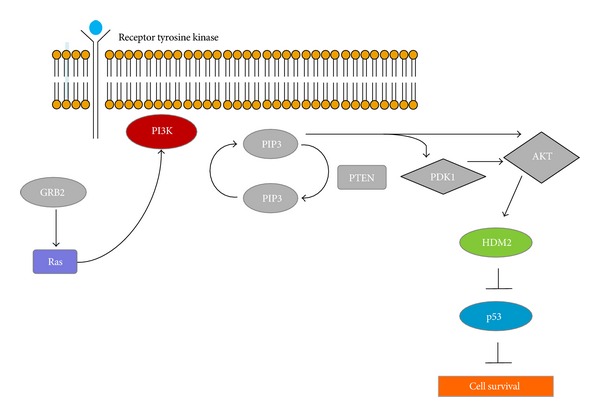
Signaling mechanism of PI3K.

Courtney and coworkers [14] described the phosphatidylinositol 3-kinase (PI3K) signaling impact on cancer cell growth, survival, and metabolism as shown in Figure 2. There are three classes of PI3K based on structure and function. Class IA PI3K is the most clearly implicated in human cancer. The MCF10DCIS cell line has been shown to have a missense mutation H1047R that maps to the kinase domain of P13K [15–17]. This substitution generates the most potently oncogenic PI3K that is known to occur with high frequency in various cancers [16]. This gain of function mutation, H1047R, is one of the “hot spot” mutations in the catalytic domain p110a of the PIK3A gene [14]. The components of the PI3K pathway are being studied as drug targets in human cancer with PI3K itself being a target for therapeutic intervention [18]. As also described in Section  3.8. Chakrabarty et al. [19] showed that the H1047R PI3K mutant enhances HER2-mediated transformation via heregulin production and activation of HER3 thereby suggesting that the PI3K H1047R mutant enhances HER2-mediated transformation by amplifying the ligand-induced signaling output in the ERB network.

### 2.3. Apoptosis

 MCF10DCIS.com cells were found to undergo spontaneous apoptosis *in vitro*, in both monolayers and spheroids [20]. Shekhar and co-workers [20] evaluated both clinically derived specimens of comedo-DCIS and MCF10DCIS cells and showed that the apoptotic pathway was associated with increased mitochondrial membrane permeability and an increase in Bax/Bcl-2 ratio occurring via a caspase-9-dependent p53-independent pathway. 

### 2.4. Metastasis

Malignant precursor cells with metastatic potential may already develop at early stages of tumorigenesis [21]. In addition, stromal cells in the microenvironment surrounding the primary tumor have been shown to be involved in facilitating metastasis [22]. Therefore, both tumor microenvironment and epithelial cells have to be considered in tumor invasion and metastasis. MCF10DCIS.com cells have been described to have a dynamic interplay of epithelium and stroma in the development of carcinoma *in situ* and subsequently in invasive ductal carcinoma (IDC) [23]. Stromal cell-derived factor-1 (SDF-1, also known as CXCL12) is a member of the CXC chemokine family [24]. SDF-1 has been identified as an estrogen-regulated gene in estrogen-receptor-(ER) positive ovarian and breast cancer cells [25]. Its effect is mediated by interaction with CXC chemokine receptor-4 (CXCR4) which is the only physiologic receptor for SDF-1 known to play a role in tumor metastasis, chemotaxis, and other metastasis components [26]. Tumors derived from the MCF10DCIS.com xenograft showed increased expression of SDF-1 in stromal cells, which is known to be highly induced by tumor-associated fibroblasts, with increased expression of CXCR4, in epithelial cancer cells during the DCIS to IDC transition [23]. This study showed that despite the phenotype of the epithelial cells being dependent upon the stroma, the malignant epithelium in these cells induced the development of the stroma which is necessary for their progression to the IDC. 

 In addition, elevated levels of MMP-2, MMP-3, MMP-9, and MMP-11 were observed in the stroma and epithelia of solid DCIS lesions prior to conversion to comedo-DCIS. The MMPs are a large family of proteases which include the stromelysins, collagenases, gelatinases, elastases, and the membrane-type MMPs. Overexpression of MMPs associated with metastasis has been reported in several cancers including breast [27]. The role of MMPs in metastasis is not only through the basement membrane (BM) and extracellular matrix (ECM) degradation but also due to the release of growth factors, such as VEGF and fibroblast growth factor (FGF), which stimulate angiogenesis. 

### 2.5. Galectin-3

Galectin-3 is a mammalian *β*-galactoside-binding protein that is expressed by various types of human cells and plays an important role in cancer cell growth, transformation, apoptosis, angiogenesis, adhesion, invasion, and metastasis [28]. The importance of Galectin-3 protein is manifested by its many effects on cancer cells. Based on a secretome study, MCF10DCIS cells were found to secrete high levels of metastatic marker Galectin-3-binding protein [11]. Amm and Buschsbaum [29] highlighted that Galectin-3 expression and genotype may be useful markers in predicting TRAIL or agonistic antibody sensitivity of breast cancer patients.

### 2.6. Osteopontin

Shevde and coworkers [30] found that when MCF10DCIS.com cells were grown in spheroids they secreted high levels of the oncogenic protein osteopontin (OPN). OPN was shown to contribute to tumorigenicity and is critical in the development of vascular-like structures in spheroids. OPN-targeting hsa-mir-299-5p was downregulated in the MCF10DCIS spheroids compared to the monolayer-derived cells. OPN is speculated to have a binding site in its 3′UTR in a region that is likely to be recognized by hsa-mir-299-5p.

## 3. SUM Breast Cancer Cell Lines

There are 11 SUM cell lines, ten of which are provided by Asterand US as shown in Table 1. Each cell line was derived from a separate individual and represents a different subtype of breast carcinoma.

**Figure 3:**
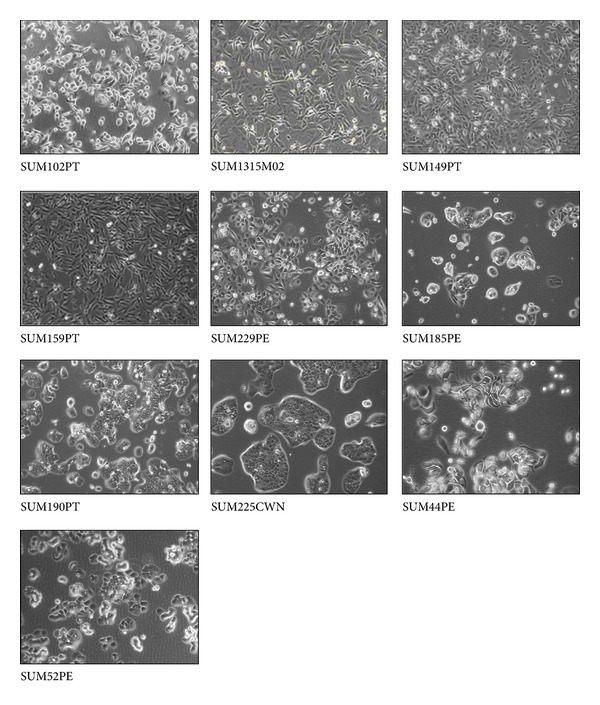
Morphology of SUM human breast cancer cell lines in culture.

The isolation and culture of these cell lines designated SUM44PE, SUM52PE, and SUM102PT have been described in detail in several publications [31–33]. Forozan et al. [34] developed seven additional human breast cancer cell lines from primary tumors. These cell lines were designated SUM149PT, SUM159PT, SUM185PE, SUM190PT, SUM225CWN, and SUM229PE. SUM206 is not available at Asterand, Inc. One cell line designated SUM1315MO2 was developed from a highly invasive breast cancer specimen that was grown for two transplant generations in immune-deficient mice before being explanted into culture. Nine of the 11 patients had received chemotherapy prior to sampling. SUM149 breast cancer cells were isolated from a patient with triple negative, inflammatory breast cancer (IBC) whose disease progressed through chemotherapy. All 11 SUM cell lines are immortal in culture with an abnormal karyotype [36] and express luminal cytokeratins 8 and 18 with the exception of SUM102PT line that expresses keratin 19. This is consistent with its basal breast epithelial cell origin [18]. Three distinct morphology groups were present among each of the SUM breast cancer cell lines, namely, epithelial cells, rounded cells, and spindle cells [35]. Keller et al. [35] classified human breast tissue through the use of a three-marker strategy into Luminal 1 cells, characterized by the majority of cells having an EpCAMhiCD24+CD49f-profile; Luminal 2 cells, characterized by a majority of EpCAMhiCD24+CD49f+ cells; basal cells, characterized by EpCAM+/loCD24-CD49f+ cells, Luminal 3 and mesenchymal cells, characterized by EpCAM-CD24-CD49f+ cells (see Section  3.7). The morphology of the SUM cell lines are shown in Figure 3 and summarized in Table 1.

### 3.1. Inflammatory Breast Cancer (IBC)

Inflammatory breast cancer (IBC) is among the most invasive, metastatic and lethal variant of human breast cancer [37]. IBC's have been reported to overexpress E-cadherin/*α*, *β*-catenin, and angiogenic features [38]. There have been very few advances in IBC-specific therapeutic targets and development of preclinical and clinical models of IBC that would enable the development of new therapeutic modalities to prolog survival of patients. The overall survival is currently 40% at three years [39]. Recently, in newly developed preclinical models of IBC and patient tumor tissues, E-cadherin, anaplastic lymphoma kinase (ALK), and HSP90 have been identified as potential targets for IBC. These targets are matched by therapeutics that are either currently in clinical trials or will be tested in clinical trials within the next year [40]. Molecular mechanisms have been implicated in IBC clinical aggressiveness and resistance to radiation [38, 40]. SUM149 and SUM190 are two cell lines established from primary IBC tumors. Both cell lines can form tumors in nude mice after mammary fat pad injection [41]. Forozan et al. [34] characterized these cell lines with respect to their ER, *TP53*, and other genes expression status. Laboratory investigations of SUM149 and SUM190 demonstrated radio resistance of these cell lines. Woodward et al. [40] used these cells as a clinical model to overcome radiation resistance to help guide clinical radiation trials against IBC. Charafe-Jauffret and coworkers [38] studied the role of cancer stem cells (CSC) in mediating metastasis in IBC and the association of these cells with patient outcome. This study suggested that the behavior of IBC could be mediated by the expression of aldehyde dehydrogenase-1 positive (ALDH1) in CSC. ALDH1 expression was the first independent prognostic marker that predicated metastasis and poor patient outcome in IBC. Victor et al. [42] investigated which proteases expressed by IBC cell lines SUM149 and SUM190 IBC cells are associated with Caveolin-1 (Cav-1) which is highly expressed in IBC patients. This protein participates in ECM degradation. Cav-1 is the major protein component of caveolac, expressed in IBC patients. Van Den Eynden and coworkers [43] showed that Cav-1 is hypomethylated and highly expressed on both IBC and SUM cell lines (SUM149 and SUM190) as opposed to the non-IBC cell line SUM 102PT. Yuan et al. [44] indicated that Cav-1 is critical for inflammatory responses regulating the STAT3/NF-*κ*B pathway. 

### 3.2. BRCA and Hormonal Status

BRCA1 and BRCA2 are human genes that belong to a class of genes known as tumor suppressors. The names BRCA1 and BRCA2 stand for breast cancer susceptibility gene 1 and breast cancer susceptibility gene 2, respectively. BRCA1 has a central role in several pathways coordinating various cellular processes in response to DNA damage, including DNA repair and preservation of genomic integrity [45]. BRCA1 and BRCA2 are also involved in pathways that regulate cell cycle progression, ubiquitylation, and apoptosis. Cells deficient in BRCA1 are unable to repair double stranded breaks [46]. BRCA1 tumors of basal-like intrinsic subtype and more frequently of medullary histology are often ER negative [47]. All the SUM cell lines of basal origin are ER and PR negative (Table 2). Luminal breast cancers are more often ER and/or PR positive or have over expression of ERBB2 [36, 48]. The epidermal growth factor receptor gene (EGFR) was found to be overexpressed without amplification in SUM102, SUM149, and SUM229 cells. However, the fibroblast growth factor receptor 1 gene (FGFR1, at11q13) is amplified in SUM44 and SUM52 cells as well as the fibroblast growth factor receptor 2 gene (FGFR2, at 10q26) is amplified in SUM-52 and SUM190. SUM225 cells have an amplified ERBB2 (encoding Her-2/*neu*). SUM1315MO2 and SUM 149PT both of basal origin were found to have BRCA1 mutations and BRCA1 allelic loss [46]. In the SUM 149PT cell line, Elstrodt and coworkers [46] identified a shift in the BRCA1 reading frame with an insertion of 12 new amino-acids after codon 723, followed by a termination codon with a 2288delT. They also identified an AG nucleotide deletion in BRCA1 at position 185 in the SUM1315MO2 cell line [48]. The 185delAG is a well described pathogenic mutation in BRCA1 and is mainly found both in familial breast and ovarian cancer among Ashkenazi Jews, though it is also found in the general population [49]. Tumors with BRCA1 mutations have also been shown to have a higher frequency of p53 mutations than sporadic breast cancers (p53 will be discussed in Section  3.4).

### 3.3. CHEK2 Mutation Analysis

**Figure 4:**
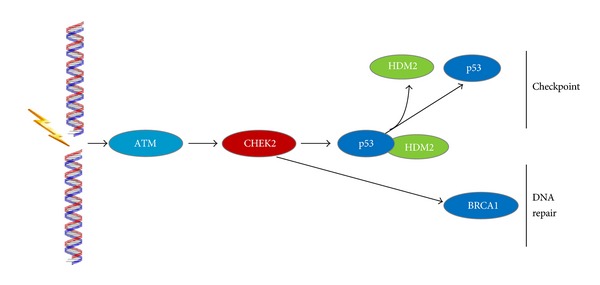
Signaling mechanism of CHEK2.

The CHEK2 gene encodes a serine/threonine kinase. CHEK2 has been described as a tumor suppressor with proapoptotic, cell cycle checkpoint, and mitotic functions (Figure 4). ATM and CHEK2 have roles upstream of BRCA1. The ataxia telangiectasia mutated protein assists cells in recognizing damaged or broken DNA strands and phosphorylates CHEK2 following DNA damage by ionizing radiation, which prevents entry of the cell into mitosis. CHEK2 then associates with phosphorylates and activates functions of BRCA1 [50].

Inherited mutations in the CHEK2 gene have been identified in some cases of breast cancer. For example, a deletionmutation at nucleotide position 1100 is associated with an increased risk of breast cancer, particularly in the European population. Association studies estimated the risk of breast cancer carriers of 1100delC to be increased by 2.7-fold in women of Northern and Eastern European descent [51, 52]. The 1100delC mutation leads to the production of an abnormally short, nonfunctional version of the CHEK2 protein. Without this protein, cells are unable to regulate cell division properly. As a result, DNA damage accumulates and cells can divide without control or order. If cell division is not tightly controlled, cancers can develop. CHEK2 is located in the center of a pathway that transduces a DNA damage signal to cellular effectors that determine response to cellular damage [53]. Wasielewski et al. [52] showed that SUM 102PT has the CHEK2 1100delC germ line founder mutation and does not express any CHEK2 protein. Furthermore, Zoppoli et al. [54] studied a panel of 60 established cancer cell lines showing that the high heterogeneity of CHEK2 expression in cancer cells is primarily due to its inactivation (low gene expression, alternative splicing, point mutations, copy-number alterations, or premature truncation) or reduction of protein levels. CHEK2, phosphorylated at T68, is also commonly activated in cancers and precancerous lesions [54]. With the exception of SUM149PT, the majority of SUM cell lines overexpress CHEK2 protein. The latter has barely detectable CHEK2 protein expression and SUM 229PE has normal expression level. SUM 44PE was not studied. 

### 3.4. Mutational Analysis and Regulation of p53 Pathway

p53 is a nuclear transcription factor and transactivates numerous target genes involved in the induction of cell cycle arrest and/or apoptosis [55]. Mutational inactivation is a major molecular mechanism of p53 dysfunction and over 50% of human cancers carry p53 mutations. Mutant p53 acts as a dominant-negative inhibitor of wild-type p53 and exhibits a longer half-life than wild-type p53 [56]. p53 truncating mutations were found in the SUM cell lines and were located throughout the p53 protein as described by Wasielewski et al. [57] and shown in Table 3. The SUM cells with truncating mutations had low transcript levels and low or no detectable protein expression levels. p53 was found to have variable transcripts and protein expression levels in SUM cell lines with p53 missense mutations (Table 3). These mutations were found in the sequence-specific DNA-binding region. This result is in concordance with the p53 missense mutations reported in clinical cancers [36].

HDM2, a negative regulator of the p53 pathway, is amplified and the protein is overexpressed in the luminal subtype SUM52PE cells [48]. HDM2 was barely detectable in the remaining SUM cell lines. SUM102PT and SUM225CWN were not analyzed. Following DNA damage, phosphorylation of HDM2 leads to changes in protein function and stabilization of p53. This occurs when HDM2 functions as an ubiquitin E3 ligase, binds to the transcriptional activation domain of p53, blocking its function, and, via ubiquitination, targets p53 for proteosome-mediated enzymatic degradation [58]. 

Another important partner of the p53 pathway is p14ARF with a role of keeping HDM2 localized in the nucleolus and preventing it from degrading p53 [59]. The induction of the p14ARF protein, the alternate reading frame product of the CDK (cyclin dependant kinase) locus, is also a mechanism that negatively regulates the p53-HDM2 interaction. p14ARF directly interacts with HDM2 and leads to upregulation of p53 transcriptional response. P14ARF therefore indirectly regulates the levels of p53. However, some evidence shows that p14ARF is able to bind p53 directly in the absence of Mdm2 [60]. The biological consequences of the p14ARF-p53 binding depend upon the cell cycle status when p53 is activated. G1 cell cycle arrest or apoptosis is observed when p53 is present and S-phase distortion is detected when p53 is inactive [61]. P14ARF is deleted in four of the five basal subtype SUM cell lines, SUM229PE, 1315MO2, SUM102PT, and SUM149PT. P14ARF showed normal transcript expression in SUM44PE, 185PE, 52PE, and 159PT (Table 3). The remaining two cell lines were not studied for this protein [48].

p21 is a potent cyclin-dependent kinase inhibitor and inhibits the activity of cyclin-CDK2 or -CDK1 complexes, and thus functions as a regulator of cell cycle progression in G_1_. The expression of this gene is tightly controlled by p53, through which this protein mediates the p53-dependent cell cycle G_1_ phase arrest in response to a variety of stress stimuli. p21 was barely detectable in 4 SUM cell lines (SUM 1315MO2, 149PT, 159 PT, and 52PE) and showed normal protein expression in the 4 SUM cell lines SUM 229PE, 185PE, 190PT, and 44PE. The remaining 2 cell lines were not analyzed [48].

### 3.5. Mutational Analysis of RB Pathway Genes

**Figure 5:**
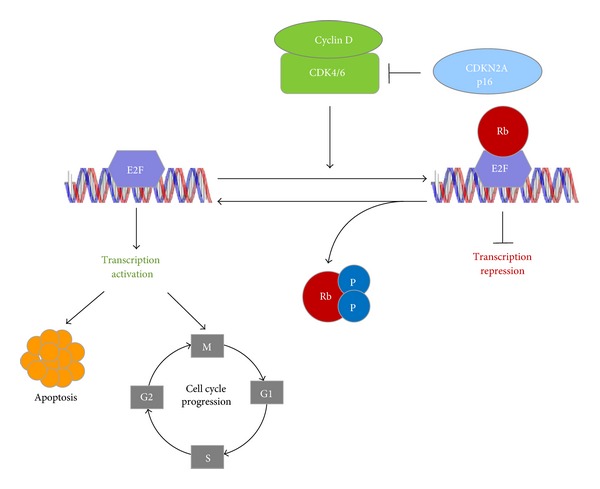
Signaling mechanism in Rb.

The Retinoblastoma protein (RB1) is a tumor suppressor that is a crucial regulator of appropriate cell cycle progression, including G_1_ to S and G_2_ to M phase transitions [62]. In cells entering the cell cycle, extracellular signals induce the expression of D-type cyclins, which bind to and activate cyclin-dependent kinases (CDK4 and CDK6) as shown in Figure 5. These complexes lead to the phosphorylation of RB1 which in turn transcriptionally activates genes required for S phase [63]. RB1 deregulation is frequently observed in multiple types of cancers [64]. RB1 is inactivated by CDK4-mediated phosphorylation, and the kinase activity of CDK4 is suppressed by p16INK4a (p16). Cyclin-dependent kinase inhibitor 2A, (*CDKN2A*, p16^Ink4A^) also known as tumor suppressor 1 (MTS-1), is a tumor suppressor protein, encoded by the *CDKN2A* gene and plays an important role in regulating the cell cycle. Mutations in p16 increase the risk of developing a variety of cancers [65]. Loss of functional p16 gives rise to unregulated CDK4 activity, leading to persistent Rb phosphorylation and uncontrolled cell proliferation [66]. Sørlie and co-workers [47] performed an extensive characterization of subtype-specific gene expression patterns at the protein and transcript level of the Rb pathway genes in SUM cells. The protein was overexpressed in SUM159PT and SUM44PE. SUM102PT and SUM44PE cells showed an overexpression of the cyclin D1 transcript and protein (Table 4).

The p16 gene was deleted in basal cell subtypes SUM102PT, SUM1315MO2, SUM149PT, and SUM229PE. The remaining three cell lines did not have detectable expression of p16 transcript or protein. No p16 deletions were found in the luminal cell subtypes. The p16 gene was found to be methylated in luminal subtype SUM 44PE and the transcript and protein levels were barely detectable (Table 4). Despite the gene being mutated in luminal subtype SUM52PE, the p16 transcripts and protein expression levels were found to be normal. Over expression or amplification of cyclin D1 (*CCND*1) is observed in as many as 50% of breast cancers, wherein it is believed to drive aberrant phosphorylation or inactivation of RB protein [67]. In breast cancer, the RB1 pathway is believed to be inactivated via several mutually exclusive mechanisms [68]. Hollestelle and coworkers [48] suggested a dichotomy in the genetic basis of human breast cancer, based on the observations whereby specific gene mutation profiles exist for the two major subtypes of breast cancer cell lines.

### 3.6. PIK3A and RAS Status (See Also Section**  **2.2)

Mutations that activated PIK3CA and RAS pathways in SUM cell lines have been identified [69] as shown in Table 5. PIK3CA was found to be constitutionally active in SUM102PT, SUM159PT, and SUM185PE, all of which possess the G12D mutation in KRAS. All the PIK3CA mutations were heterozygous with the exception of the H1047R mutation found in SUM185PE [69]. SUM159 had an H1047L mutation in P1K3CA (Table 5) and a G12D mutation in HRAS. SUM 229PE had wild-type P1K3CA. Since activating RAS isoforms have been reported for KRAS, HRAS and NRAS, Ras mutations have been identified in many tumor types and show tissue specificity [70].

### 3.7. E-Cadherin/*α*-, *β*- and *γ*-Catenin Expression Pattern of Asterand SUM Cell Lines

 The E-cadherin/catenin protein complex maintains the integrity of epithelial tissues through cell-cell adhesion [71, 72]. The E-cadherin/catenin protein complex consists of the cytoplasmic proteins *α*-catenin, *β*-catenin, *γ*-catenin, p120-catenin, and the transmembrane protein E-cadherin. Extracellular E-cadherin forms homodimers with E-cadherin proteins on adjacent epithelial cells in a calcium-dependent manner. Decreases in this adhesion ability of the cell have been linked to metastasis and tumor progression [73].

Hollestelle and colleagues [74] used human breast cancer cell lines that included all SUM cells to study E-cadherin inactivation. E-cadherin and *α*-, *β*-, and *γ*-catenin protein expressions were determined by immunohistochemistry. E-cadherin gene status was analyzed for gene mutations and promoter methylation status. All the spindle shaped, basal morphology SUM cell lines showed promoter hypermethylation indicating inactivation of E-cadherin. SUM1315MO2 had barely detectable *γ*-catenin protein expression and no E-cadherin expression. SUM159PT also did not show E-cadherin protein expression. SUM44PE with luminal subtype and rounded morphology had a protein truncating mutation in the E-cadherin gene with no detectable E-cadherin or *α*-catenin and barely detectable *γ*-catenin protein expression. 

### 3.8. Expression of CK8/18/19 and CD49f after CD146/EpCAM Enrichment of SUM Cell Lines

Epithelial cell adhesion molecule (EpCAM) also known as CD326 is a type I transmembrane 39–42 kDa glycoprotein that functions as a homophilic, epithelial-specific intercellular cell-adhesion molecule. The 314 amino acid-long EpCAM protein comprises a large extracellular domain with an epidermal growth-factor- (EGF-) like domain and a putative thyroglobulin (TY) domain, a single transmembrane region, and a short (26 amino acids) cytoplasm tail [75]. Recent data suggest that EpCAM's role is not limited to cell adhesion, but it is also involved in cellular signaling, cell migration, proliferation, and differentiation [76]. EpCAM's association with proliferation, adhesiveness, tissue stabilization, promotion of tumor growth, and metastasis suggests that EpCAM is a pleiotropic molecule that potentially offers therapeutic applications in cancer treatment [53]. Mostert and coworkers [77] undertook a clinical study in primary breast cancer patients in which both EpCAM and CD146-positive circulating tumor cells (CTCs) were studied to establish the clinical relevance of cell lines including the SUM cell lines. CD146 was highly expressed in SUM1315MO2 and SUM159PT with epithelial-mesenchymal transition (EMT) features (as shown in Table 1). In addition to examining the added value of selecting cancer cells with a broader array of cytokeratins (CKs), they tested CD49f (ITGA6; integrin, alpha 6) as an alternative selection marker for CTC. CD49f is also an integral cell-surface protein involved in cell adhesion which has also been described as a stem cell marker in breast cancer [78], making it a candidate to select EMT-like breast cancer cells with stem cell-like features. Mostert and colleagues [77] set out to identify an additional new marker to be used after capturing breast cancer cells with combined anti-CD146/anti-EpCAM, to detect those cancer cells that lack CK8/18/19 expression.  Table 6 summarizes their work in which SUM159PT was the only SUM cell line negative for these markers. 

Interestingly, some SUM cell lines express markers of some of cancer stem cell (CSC) populations namely, ALDH, CD44+, and CD24− [35, 84]. ALDH is a detoxifying enzyme responsible for the oxidation of intracellular aldehydes and is thought to play a role in stem cell differentiation via metabolism of retinol to retinoic acid [78]. Breast carcinomas have been reported to contain a subpopulation of CD44+/CD24− tumor cells with stem cell-like properties. The subpopulation of breast cancer cells with CD44+/CD24− has been shown to exhibit enhanced invasive phenotype as an early step to metastasis [35, 85].

The phenomenon of heterogeneity has also been well documented in breast cancer [86]. This has been attributed to the presence of cancer stem cells (CSC's) that have the properties of differentiating along divergent pathways. The percentage of cancer cells that express CD44+/CD24− phenotype correlate with spindle/mesenchymal physical features [87]. CD44, a multifunctional class I transmembrane glycoprotein, generally acts as a specific receptor for hyaluronic acid, promoting migration in normal cells and is highly expressed in almost every cancer cell [88]. CD44 is mainly associated with proteins that monitor the extracellular changes and plays a critical role in regulating cell adhesion, proliferation, growth, survival, motility, migration, angiogenesis, and differentiation [89–91]. CD24 is a small cell-surface protein molecule anchored by glycosyl-phosphotidyl inositol in a wide variety of cancer cells. It is heavily glycosylated and functions in cell-cell and cell-matrix interactions [91]. CD24 is another important marker whose prognostic value and significance remains controversial [92, 93]. CD24 is highly expressed in ovarian, breast, prostate, bladder, renal, non-small cell carcinomas, and other human cancers and is involved in cell adhesion and metastasis [92]. This indicates that CD24 could be a significant marker in tumor prognosis and diagnosis. The metastatic association of CD24 increases its importance as a prognostic factor and a CSC marker [90]. Marotta and coworkers [94] targeted the JAK2/Stat3 signaling pathway for specific breast cancer therapies by highlighting the difference between distinct breast cancer cell types. They found that the IL-6/JAK2/Stat3 pathway was preferentially active in CD44+ CD24− breast cancer cells compared to other tumor cell types.

Subpopulations of SUM149PT and SUM159PT cells, selected for a given phenotypic state both luminal and basal cells, were shown to have the ability to give rise to stem cells [95]. SUM149PT and SUM159PT cells were treated with either paclitaxel or 5-fluorouracil *in vitro* and showed an increased number of stem cells in both cell lines. The authors used the Markov mathematical model in which cells transition stochastically between states to predict the conversions that were most likely to have occurred to achieve this outcome. 

### 3.9. Clinical Relevance of Markers and Targets Aberrantly Expressed in MCF10DCIS and SUM Cell Lines

 Mutations affecting the coding sequence in these cell lines are reported in the COSMIC database and other resources are shown in Table 7. Proia and coworkers [96, 97] reported that basal breast epithelial cells derived from patients and cell lines SUM1315 MO2  and harboring deleterious mutations in BRCA1 (BRCA1mut/+) give rise to tumors with increased basal differentiation relative to cells from BRCA1+/+ patients.

The PI3K  pathway is one of the most important pathways in cancer metabolism and growth. Mutations in the PI3K  pathway are frequent in breast cancer and result in resistance to HER2-targeted agents hormonal agents [98]. The PI3K  inhibitors XL147 and XL765 developed by Exelixis and Sanofi-aventis have been shown to effectively block the PI3K  and ERK pathways in Phase I studies [98]. Phase II clinical trials with XL147 in hormone-refractory disease and also in patients who have failed trastuzumab (trade name Herceptin), are currently in progress [99]. 

Hormone receptor-positive breast cancer therapies have been the main drug targets for several decades. HER2 became an accepted therapeutic target in standard breast cancer practice. Trastuzumab, a monoclonal antibody that interferes with the HER2/neu receptor, has been developed by Genentech. In addition to the treatment with trastuzumab other approaches have been developed [100, 101]. One approach has been the development of HER2-directed antibody-drug conjugates. Trastuzumab-DM1 (T-DM1) is an anti-HER2 antibody-drug conjugate consisting of trastuzumab covalently bound via a linker to DM1, a derivative of the antimicrotubule chemotherapy maytansine. Patient cohorts with HER2-positive breast cancer that progressed during trastuzumab therapy and developedresistance to the treatment were assignedto the clinical trials with T-DM1. A significantclinical benefit among patients treated at the recommended dose of T-DM1 was observed in Phase I studies [102]. This single-agent activity was subsequently confirmed in a Phase II study in another cohort of trastuzumab-resistant patients [103]. 

Recently, HER3 and its physiologic ligand heregulin (HRG) have been implicated in the development of resistance to antiestrogen therapies [102]. Presently, a humanized anti-HER3 monoclonal antibody MM-121 is available. This antibody binds to HER3 and prevents phosphorylation of HER3 and also effectively inhibits the HER2/HER3 heterodimer [104]. This compound is in Phase II studies, in combination with the nonsteroidal AI exemestane, in patients with advanced breast cancer that had previously progressed on endocrine therapies [100]. 

Triple-negative breast cancer (TNBC) is an aggressive disease with about 70% of breast tumors falling within the basal-like group of breast cancers [105]. A large majority of women with germline *BRCA1 *mutations have the triple-negative phenotype clustered within the basal group [101]. In 2009, Iglehart and Sliver [106] showed that inhibiting the remaining DNA repair machinery within BRCA-deficient cancer cells using (PARP) poly ADP ribose polymerase inhibition results in synthetic lethality. In 2011, AstraZeneca performed a proof of principal clinical trial on 54 subjects with *BRCA1 *or *BRCA2 *mutations with advanced breast cancer that were treated with PARP inhibitor olaparib [107]. Oral olaparib was administered to patients without significant toxicity. In addition, survival improvement was observed when Sanofi performed a randomized Phase II trial among patients with TNBC using another PARP inhibitor, iniparib (BSI-201) combined with carboplatin and gemcitabine [108]. The Hoosier Oncology group has currently an active Phase II [109] ongoing with a PARP inhibitor to evaluate 2-year disease-free survival in patient population treated with single-agent Cisplatin and patients treated with Cisplatin in combination with Rucaparib, another PARP inhibitor following preoperative chemotherapy treatment. The Lee Moffitt Cancer Center and Research Institute also has currently an active phase I/II clinical trial in progress [110] studying the Ad.p53 DC Vaccine and 1-MT in metastatic invasive breast cancer. Based on a report published by Bioseeker group (BSG) in October 2012 there are over 350 companies developing over 479 drugs targeting breast cancer. They identified drugs that are linked to more than 247 different targets which are divided into 61 classifications of molecular function.

Recently the cancer genome network (TCGA) reported a comprehensive molecular analysis of breast cancer tumors with a catalogue of likely genomic drivers of the most common breast cancer subtypes [111]. They categorized breast cancer into four main phenotypic cancer classes to explain the phenotypic heterogeneity observed within defined breast cancer subtypes. They identified two novel protein-expression-defined subgroups by identifying specific signaling pathways dominant in each molecular subtype including a HER2/phosphorylated HER2/EGFR/phosphorylated EGFR signature within the HER2-enriched expression subtype. Anti-EGFR (epidermal growth factor receptor) therapies, including tyrosine kinase inhibitors (TKIs) and monoclonal antibodies, are available and could potentially be used in breast cancer therapy.

## 4. Conclusions

This is a summary of several studies performed over the years that reveal the signaling pathways governing the MCF10DCIS.com and SUM cell lines. Breast tumors can be classified by their intrinsic subtypes. These subtypes have been associated with differences in survival with the basal-like and HER2-positive subtypes having the shortest survival times [112]. There are major gene pathways that give rise to the nature of MCF10DCIS.com and the SUM cells. This paper will serve as a valuable resource for future research in the identification of biomarkers and drug discovery, in breast cancer. Another important resource summarized in Table 7 is a list of mutation affecting the coding sequence in these cell lines as reported in the COSMIC database [83]. 

As we complete this paper, there are 538 clinical trials focused on breast cancer currently in progress. The breast cancer clinical trials are categorized either by type of breast cancer such as inflammatory or triple negative breast cancer or by response and resistance to treatments [113]. Most of these trials are not stratified by the analysis of individual tumor molecular marker profiles or mechanistic hypothesis. Although a signature specific to a specific subtype may not predict overall outcome, such a signature would have both biological and clinical utility. The utilization of highly characterized cells in the R&D will aid in the development of highly specific therapy and patient stratification. 

## Figures and Tables

**Table 1:** Cell source and clinicopathological characteristics SUM cell lines provided by Asterand.

	Cell line	Cell source	Cellular morphology	Clinicopathology
1	SUM102PT	Intraductal carcinoma/microinvasion	nd	Basal B
2	SUM1315MO2	Skin metastasis of infiltration ductal carcinoma	Spindle	Mesenchymal
3	SUM149PT	Invasive ductal carcinoma (inflammatory)	Mixed	Basal B
4	SUM159PT	Anaplastic carcinoma	Spindle	Mesenchymal
5	SUM229PE	Pleural effusion	Spindle	Basal B
6	SUM185PE	Pleural effusion	nd	Luminal
7	SUM190PT	Invasive ductal carcinoma (inflammatory)	nd	Luminal
8	SUM225CWN	Chest wall recurrence of ductal carcinoma *in situ *	Epithelial	Luminal
9	SUM44PE	Pleural effusion	nd	Luminal
10	SUM52PE	Pleural effusion	nd	Luminal

Source: [31–35]; nd = not determined.

**Table 2:** ER, PR, and BRCA1 profile of SUM cell lines.

Cell line	BRCA1			ER protein	PR protein	ERBB2
	Allelic loss	Mutation	Protein effect			
SUM102PT	No loss	Wild type	NA	nd	nd	
SUM1315MO2	**Loss**	**185del AG**	**truncation **	−	−	
SUM149PT	**Loss**	**2288delT**	**truncation **	−	−	
SUM159PT	No loss	Wild type	NA	−	−	
SUM229PE	No loss	Wild type	NA	−	−	
SUM185PE	**Loss**	Wild type	NA	−	−	
SUM190PT	**Loss**	Wild type	NA	−	−	**+++**
SUM225CWN	**Loss**	Wild type	NA	−	−	**+++**
SUM44PE	**Loss**	Wild type	NA	**+++**	**+++**	
SUM52PE	**Loss**	Wild type	NA	**+++**	−	

Source: [36, 46, 48]; ER: estrogen receptor, PR: progesterone receptor, NA: not applicable; nd: not determined, −: no expression, +++: overexpression, +: normal expression.

**Table 3:** p53 profile of the Asterand SUM cell lines.

Cell line	17p allelic loss	p53 gene sequence				p53 expression		p14ARF status	
		Exon	Mutation	Amino acid change	Protein effect	Transcript size (kb)	Transcript expression	Gene	Transcript expression
SUM102PT					Wild type		nd	**Deleted **	−
SUM1315MO2	Loss	5	TGC>TTC	C135F	**Missense **	**1**	**+ **	**Deleted **	−
SUM159PT	Loss	5	TCC>TCCTCC	S158insS	**Missense**	**0.7**	−		+
SUM149PT	Loss	7	ATG>ATA	M237I	**Missense **	**1**	**+ **	**Deleted **	−
SUM225CWN	Loss	8	CTG>CCG	L265P	**Missense **	**0.9**	**++ **	nd	nd
SUM229PE	Loss	8	CGT>TGT	R273C	**Missense **	**0.7**	nd	**Deleted **	−
SUM44PE	Loss	3	GAA>CA	E28fsX16	**Truncating **	**0.4**	−	wt	**+ **
SUM185PE	Loss	5	CAG>TAG	Q144X	**Truncating **	**0**	−	wt	**+ **
SUM52PE	Loss	6	CGA>TGA	R213X	**Truncating **	**0.3**	−	wt	**+ **
SUM190PE	Loss	9	CAG>TAG	Q317X	**Truncating **	**0.4**	±	nd	nd

Source: [36, 46, 52], +: normal expression, −: not detectable, nd: not determined, wt: wild type, ±: barely detectable, ++: overexpression.

**Table 4:** Mutational analysis Rb pathway genes for Asterand SUM cell lines.

Cell line	RB1 protein expression	Cyclin D1 transcript expression	Cyclin D1 protein expression	p16 gene sequence	p16 transcript expression	p16 protein expression
SUM102PT	nd	**++**	nd	**Deleted**	**+**	**nd**
SUM1315MO2	**+ **	**+**	±	**Deleted**	−	−
SUM149PT	**+ **	**+**	±	**Deleted**	−	−
SUM159PT	**+ + **	**+**	**+**	Wild type	−	±
SUM229PE	**+ **	**+**	**+**	**Deleted**	−	−
SUM185PE	**+ + **	**+**	**+**	Wild type	±	±
SUM190PT	nd	nd	nd	nd	nd	nd
SUM225CWN	nd	nd	nd	nd	nd	nd
SUM44PE	**+ **	**++**	**++**	**Methylated**	±	±
SUM52PE	**+ **	**+**	**+**	**Mutant**	**+**	**+**

Source: [48]; nd: not determined, +: normal expression, ±: barely detectable, −: no detectable expression, ++: over expression.

**Table 5:** PIK3CA, HRAS and KRAS mutation status for SUM cell lines.

Cell line	PIK3CA status		HRAS status		KRAS status	
	Gene sequence	Amino acid change	Gene sequence	Amino acid change	Gene sequence	Amino acid change
SUM102PT	3140A>G	H1047R	wt	None	wt	None
SUM1315MO2	wt	None	wt	None	wt	None
SUM149PT	wt	None	wt	None	wt	None
SUM159PT	**3140A>T**	**H1047L **	**35G>A **	**G12D **	**wt**	**None**
SUM229PE	wt	None	wt	None	**35G>A **	**G12D **
SUM185PE	**3140A>T**	**H1047R **	wt	None	wt	None
SUM190PT	**3140A>G**	**H1047R **	wt	None	wt	None
SUM225CWN	wt	None	wt	None	wt	None
SUM44PE	wt	None	wt	None	wt	None
SUM52PE	wt	None	wt	None	wt	None

Source: [69]; +: normal expression; —: not detectable, nd: not determined, wt: wild type.

**Table 6:** Protein expression of cytokeratins and CD49f/EPCAM in SUM cells.

Cell lines	CK5 (KRT5)	CK14 (KRT14)	CK8-18	CK19 (KRT19)	CD49f (ITGA6)	CD146 (MCAM)	EpCAM (TACSTD1)
	IHC	IHC	IHC	IHC	IHC	FACS (s/n)a	FACS (s/n)a
Normal like							

SUM1315MO2	+	−	nd	nd	+	20–200	<5
SUM159PT	−	−	−	−	+++	20–200	<5
SUM102PT	nd	−	nd	nd	nd	520	<5

Basal							

SUM149PT	++++	−	++++	++	+	20–200	20–200
SUM229PE	+++	−	++++	++	+	20–200	20–200

Luminal							

SUM44PE	−	−	nd	+++	++++	nd	nd
SUM185PE	−	−	++++	+++	++++	nd	nd

Source: [35, 77].

**Table 7:** Summary of mutations affecting the coding sequence of Asterand breast cancer cell lines.

Coding mutations listed in COSMIC database						
Cell line	Gene	AA mutation	CDS mutation	Mutation	Zygosity	Reference
MCF10DCIS	PI3KCA	c.3140A>G	p.H1047R	Missense	Unknown	Kalaany and Sabatini (2009) [17]
SUM102PT	CDKN2A	471 del 471	None	Whole gene deletion	Homozygous	Hollestelle et al. (2010) [48]
	PIK3CA	c.3140A>G	p.H1047R	Missense	Heterozygous	
SUM1315MO2	BRCA1	66_67 del AG	p.E23fs*17	Frameshift	Homozygous	Hollestelle et al. (2010) [48]
	CDKN2A	471 del 471	None	Whole gene deletion	Homozygous	
SUM149PT	FBXW7	c.1644 1645 ins 416	F549Fs*6	Frameshift	Homozygous	Strohmaier et al. (2001) [79]
	BRCA1	c.2169 del T	p.P724fs*12	Frameshift	Homozygous	Hollestelle et al. (2010) [48]
	CDKN2A	471 del 471	None	Whole gene deletion	Homozygous	
	EP300	c.4025+(28)del3	unknown	Unknown	Heterozygous	Gayther et al. (2000) [80]
SUM159PT	HRAS	c.35G>A	p.G12D	Missense	Heterozygous	Hollestelle et al. (2007) [69]
	PIK3CA	c.3140A>T	p.H1047L	Missense	Heterozygous	
SUM229PE	KRAS	c.35G>A	p.G12D	Missense	Heterozygous	Hollestelle et al. (2010) [48]
	CDKN2A	471 del 471	None	Whole gene deletion	Homozygous	
SUM185PE	PIK3CA	3140A>G	p.H1047R	Missense	Heterozygous	Saal et al. (2005) [81]
SUM190PT	PIK3CA	3140A>G	p.H1047R	Missense	Heterozygous	Hollestelle et al. (2007) [69]
SUM225CWN	None					
SUM44PE	CDH1	1269delT	F423fs*8	Frameshift	Homozygous	Van de Wetering et al. (2001) [82]
SUM52PE	CDKN2A	203C>T	A68V	Missense	Homozygous	Hollestelle et al. (2010) [48]

Source: [83].
